# 5-Methylcytosine and 5-Hydroxymethylcytosine Spatiotemporal Profiles in the Mouse Zygote

**DOI:** 10.1371/journal.pone.0038156

**Published:** 2012-05-31

**Authors:** Juliette Salvaing, Tiphaine Aguirre-Lavin, Claire Boulesteix, Gaëtan Lehmann, Pascale Debey, Nathalie Beaujean

**Affiliations:** 1 INRA, UMR1198 Biologie du Développement et Reproduction, Jouy-en-Josas, France; 2 ENVA, Maisons Alfort, France; Université Paris-Diderot, France

## Abstract

**Background:**

In the mouse zygote, DNA methylation patterns are heavily modified, and differ between the maternal and paternal pronucleus. Demethylation of the paternal genome has been described as an active and replication-independent process, although the mechanisms responsible for it remain elusive. Recently, 5-hydroxymethylcytosine has been suggested as an intermediate in this demethylation.

**Methodology/Principal Findings:**

In this study, we quantified DNA methylation and hydroxymethylation in both pronuclei of the mouse zygote during the replication period and we examined their patterns on the pericentric heterochromatin using 3D immuno-FISH. Our results demonstrate that 5-methylcytosine and 5-hydroxymethylcytosine localizations on the pericentric sequences are not complementary; indeed we observe no enrichment of either marks on some regions and an enrichment of both on others. In addition, we show that DNA demethylation continues during DNA replication, and is inhibited by aphidicolin. Finally, we observe notable differences in the kinetics of demethylation and hydroxymethylation; in particular, a peak of 5-hydroxymethylcytosine, unrelated to any change in 5-methylcytosine level, is observed after completion of replication.

**Conclusions/Significance:**

Together our results support the already proposed hypothesis that 5-hydroxymethylcytosine is not a simple intermediate in an active demethylation process and could play a role of its own during early development.

## Introduction

DNA methylation, and epigenetic modifications in general, play a major role during early embryonic development, in particular embryonic genome activation, X inactivation, differentiation [1], [2]… DNA methylation levels undergo major modifications that appear to be essential for early development [3], [4] and necessary for the establishment of a pluripotent state by the demethylation of many pluripotency regulators [5]. DNA methylation changes during early embryonic development have been studied in many species. One of the main features, observed primarily in mice, is the asymetric dynamics between the paternally and maternally inherited parts of the genome in 1-cell embryos just after fertilization [6]–[10]. Indeed, in mouse embryos, using a 5-methylcytosine (5MeC) antibody, a very rapid demethylation was observed in the paternal genome (constituting the paternal pronucleus), prior to the onset of replication [6]–[8], while progressive demethylation in the maternal one occurs across the following cell-cycles until the morula stage [6]–[8], [11]. Demethylation in the paternal pronucleus has thus been called “active” as opposed to the “passive” demethylation observed in the maternal pronucleus. Passive demethylation results from a dilution of the original methylation pool, due to the absence of methylation maintenance during replication [11], [12]. Indeed, DNA methylation is ensured by enzymes called DNA methyl-transferases (Dnmt), which include the maintenance methyl-transferase Dnmt1, involved in copying DNA methylation patterns on the newly synthetised strand during replication, and the *de novo* methyl-transferase Dnmt3A and 3B [13], that establish newly methylated domains.

Active demethylation has been observed on a large scale only in embryos and in primordial germ cells [14], and the mechanism sustaining it remains largely unknown, even if some advances in this domain have been published lately [15]–[17]. Last year, the elongator complex has been suggested to play a role in DNA methylation, but it remains unclear whether that role is direct or indirect [15], and probably the most prominent mechanism proposed so far involve DNA repair pathways [16], [17].

However, the recent rediscovery of 5-hydromethylcytosine (5hMeC) [18], [19] has led to new speculations about the function of this mark as an intermediate in the DNA methylation pathway. Recent studies in the mouse embryo have shown a good complementarity between 5MeC and 5hMeC levels, the latter increasing in the paternal pronucleus when 5MeC decreases [12], [20]–[23]. Additionally, while rings of 5MeC persist around the nucleolar precursor bodies (NPBs) in the paternal pronucleus [24], similar rings were observed for 5hMeC in the maternal pronucleus [21]. Since the localization of those rings strongly reminds that of pericentric heterochromatin [25]–[27], it has been proposed that 5MeC and 5hMeC complement each other, the first one marking the paternal heterochromatin, while the second one marks the maternal heterochromatin. 5hMeC can be further converted to 5-carboxylcytosine (5caC) and to 5-formylcytosine (5fC) [28], [29], both of which can be detected in the mouse zygote [30]. In cells, 5caC can be excised by the thymine-DNA glycosylase [29] while in embryos 5caC and 5fC appear to be lost by progressive dilution [30]. However, 5hMeC persists much longer than it would be expected for a simple intermediate in DNA demethylation [20] and seems to be removed during preimplantation development by a passive dilution mechanism similar to the one observed for 5MeC in the maternal pronucleus [12]. In addition, as the levels of 5hMeC remain high in undifferentiated cells and are lost only upon differentiation [22], [31], it raises the possibility that 5hMeC might have an additional function in pluripotent stem cells as well as in the totipotent early embryo.

In order to investigate in more details the spatiotemporal relationship between 5MeC and 5hMeC patterns during the first cell cycle in the early mouse embryo, we examined the colocalization of 5MeC, 5hMeC and pericentric heterochromatin, using immunoFISH experiments. In a second part, we performed a quantitative analysis of 5MeC and 5hMeC dynamics during the replication phase, using labelled deoxyuridine incoporation, which allows precise timing of replication [32], [33]. All together, our results show that the complementarity between 5MeC and 5hMeC is not as perfect as it seems at first glance.

## Results

### Methylation and hydroxymethylation of pericentric heterochromatin

Previous studies described complementary patterns of 5-methylcytidine (5MeC) and 5-hydroxymethylcytidine (5hMeC) both in terms of expression levels as in terms of localization [20], [21]. In particular, a persisting ring of 5MeC was observed in the paternal pronucleus around the nucleolar precursor bodies (NPB) [24], while a similar ring of 5hMeC was shown around the NPB in the maternal pronucleus [20], [21]. The authors proposed that those rings correspond to the pericentric heterochromatin, which shows a very similar localization [25], [26], but they did not provide any direct evidence. We therefore performed immunoFISH experiments on 1-cell stage embryos with a preserved 3D organization [27], using an antibody against 5MeC or an antibody against 5hMeC, together with specific probes for the major satellites (pericentric DNA). Embryos were fixed at different time points (19, 21, 23, 25 and 29 hphCG), allowing us to observe them at all the stages of the first cell cycle, from fertilization up to the first mitosis. We used the PN classification from Adenot [33] to sort the embryos.

**Figure 1:**
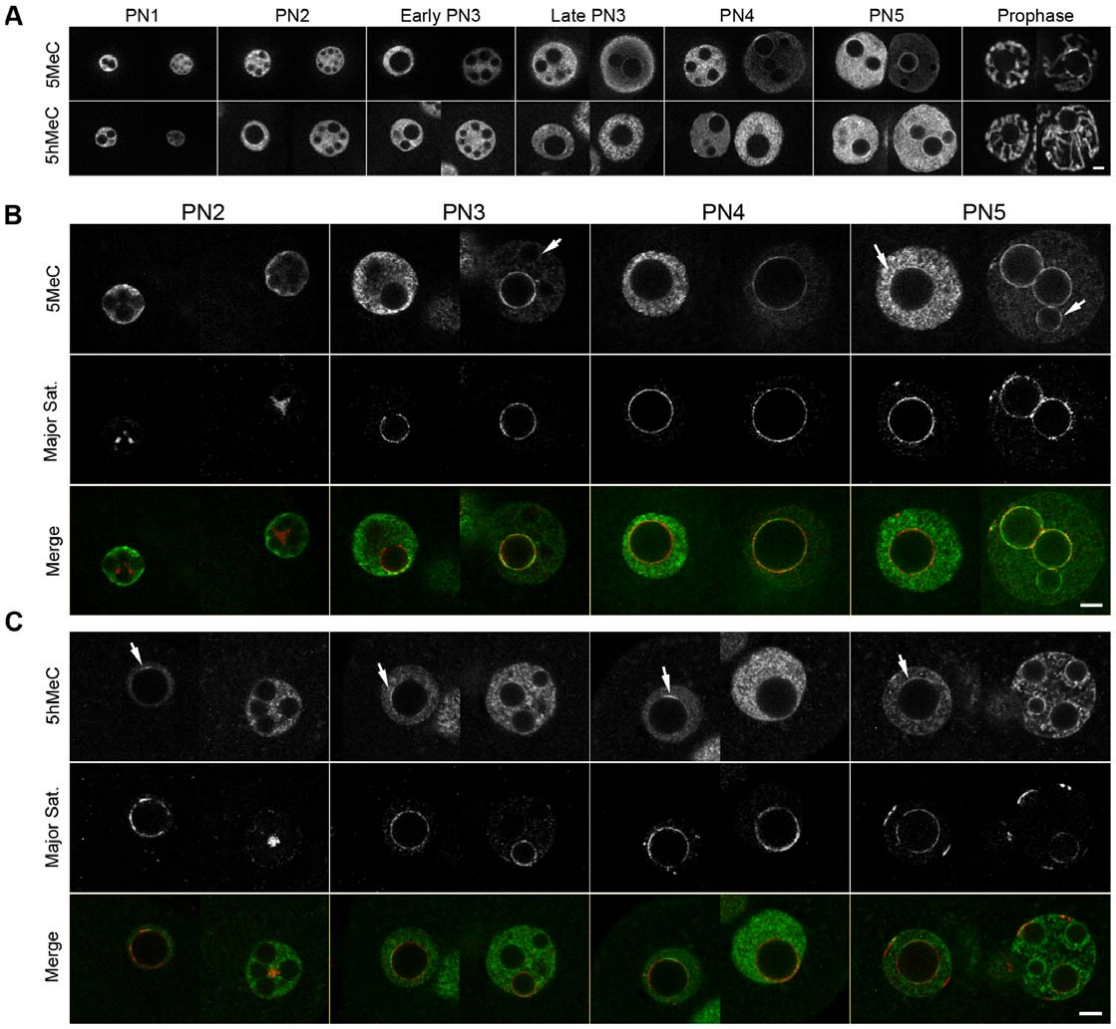
DNA methylation and hydroxymethylation on pericentric heterochromatin. Z-stack images of 1-cell embryos (PN1 to Prophase) were acquired in 3D but only single representative z-sections for each PN are shown here. Images were rotated if necessary to have the maternal PN on the left and the paternal PN on the right. A) 3D immunostainings using either an anti-5-methylcytosine antibody (n = 148, upper row) or an anti-5-hydroxymethylcytosine antibody (n = 226, lower row). B) 3D immunoFISH using an anti-5MeC antibody (upper row) and specific probes for the major satellites (middle row). Merged images are shown on the lower row (green: 5MeC; red: major satellites) ; a total of 75 embryos were analyzed C) 3D immunoFISH using an anti-5hMeC antibody (upper row) and specific probes for the major satellites (middle row). Merged images are shown on the lower row (green: 5hMeC; red: major satellites); a total of 61 embryos were analyzed. Scale Bar: 5 µm.

As expected, 5MeC is found preferentially in the maternal pronucleus while 5hMeC is more strongly expressed in the paternal one (figure 1). It is however important to note that we could always detect a signal for 5MeC and 5hMeC respectively in the paternal and maternal pronuclei. It should also be mentioned that we did not observe any strong differences between the 5MeC and 5hMeC signals obtained with immunoFISH and those obtained with classical immunostainings (figure 1A vs 1B/C).

As shown on figures 1B and C, in the maternal pronucleus major satellites remain localized around the NPBs, with sometimes a few signals at the periphery, from PN1 until the end if the 1-cell stage (figure 1 B and C). We could not detect any accumulation of 5MeC on these, except for a very weak and partial accumulation sometimes observed at the PN5 stage (figure 1B; arrow). On the other hand, we observed accumulations of 5hMeC, in partial rings around the NPBs or sometimes as spots near the nuclear periphery, from PN2 until mitosis (figure 1C; arrows). However, only a small fraction of the major satellites signals clearly colocalizes with 5hMeC, suggesting that it accumulates on the pericentric heterochromatin of a subset of chromosomes only.

In the paternal pronucleus, we could not detect any strong accumulation of 5MeC on the major satellites during the early stages (until early PN3; figure 1B). During that time, the major satellites remain mostly in clusters, and organize around the NPBs at the PN3 stage only (figure 1 B and C). Once the rings are formed, a very strong 5MeC signal colocalizing with the major satellites signal is observed. Interestingly, at the PN3 and PN4 stages, 5MeC signal is very strong around the NPBs surrounded by major satellites, with an almost perfect colocalization; also, a very faint signal is sometimes observed around the other NPBs (arrow). Finally, at the PN5 stage, 5MeC rings are clearly observed around all NPBs, even in the absence of pericentric DNA (arrow). In contrast to 5MeC, there is no accumulation of 5hMeC signal on the pericentric heterochromatin regions at PN2 and 3 (figure 1C). However, faint and partial rings start to be visible around the NPBs at PN4, and at PN5 all the NPBs, including those not surrounded by pericentric heterochromatin, are surrounded with strong 5hMeC signal. There is therefore no exclusion of 5MeC and 5hMeC on the pericentric heterochromatin at the end of the first cell stage.

### DNA methylation, hydroxymethylation and Replication

**Figure 2:**
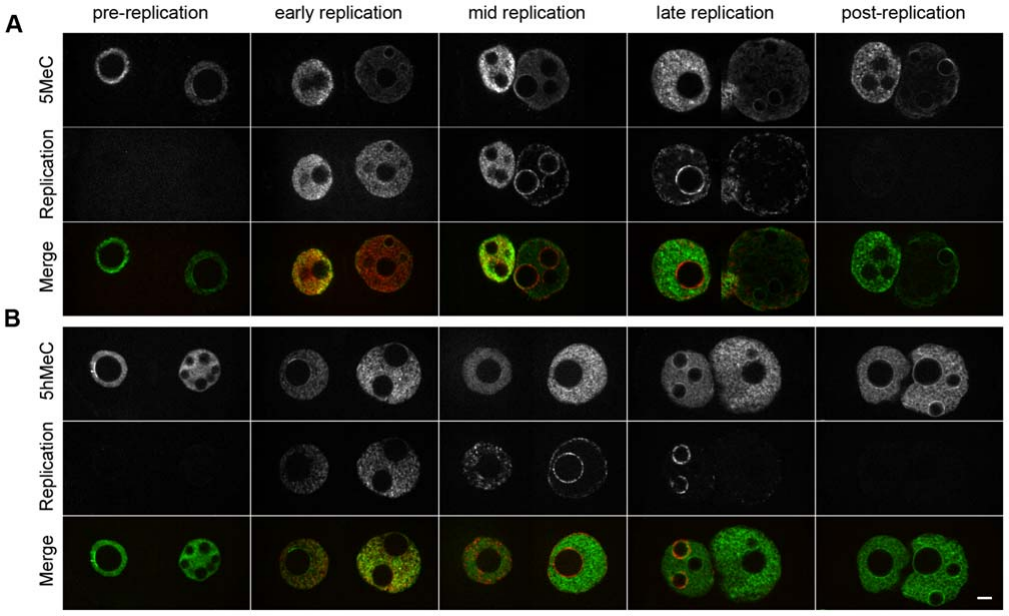
DNA methylation and hydroxymethylation patterns during replication. Representative z-section images of maternal PN (left) and paternal PN (right) in 1-cell embryos with various replication statuses: from pre-replication to post-replication. A) Double immunostaining of 5MeC (upper row) and DIG dUTP (middle row). Merged images are shown on the lower row (green: 5MeC; red: replication); a total of 89 embryos were analyzed. B) Double immunostaining of 5hMeC (upper row) and BrdU (middle row). Merged images are shown on the lower row (green: 5MeC; red: replication); a total of 226 embryos were analyzed. Scale Bar: 5 µm.

Since DNA methylation and hydroxymethylation patterns are not fully complementary on the spatial level, we wanted to adress their complementarity on the temporal level. The replication in particular is a very interesting period: while DNA methylation has been described as replication-independent and complete before the start of replication [7], recent studies [12], [20], [21] as well as our own observations (figure 1A) have shown changes in the methylation levels up to PN4 or PN5, that is after the beginning of replication [32], [33]. Double stainings for replication and either 5MeC or 5hMeC were therefore performed. Embryos were sorted in 5 different classes (Table 1), based on the previous work from Bouniol-Baly and collaborators [32]. Those without any replication staining were called “pre-replication” or “post-replication”. Embryos with homogeneous replication staining in both pronuclei were classified as “early replication”, those with homogeneous replication staining in the maternal pronucleus but peri-NPB staining in the paternal one as “mid replication”, and those with peri-NPB staining in the maternal pronucleus and peri-nuclear staining in the paternal one as “late replication” (figure 2).

**Table 1:** Status of replication according to the PN stage and time of fixation.

	Pre	Early	Mid	Late	Post
Early PN3 (20–21 hphCG)	**23 (100%)**				
PN3 (21–23 hphCG)	10 (16.9%)	**43 (72.9%)**	4 (6.8%)	1 (1.7%)	1 (1.7%)
Early PN4 (23–24 hphCG)		2 (6.9%)	**11 (37.9%)**	**14 (48.3%)**	2 (6.9%)
Late PN4 (25–26 hphCG)			3 (13%)	5 (21.7%)	**15 (65.2%)**

Embryos were first sorted according to their PN stage and the timing of their fixation. Then, for each category, embryos were sorted again according to their replication staining. Early PN3 embryos are exclusively in pre-replication, PN3 are in majority in early replication, early PN4 are mostly in mid/late replication and most of the late PN4 have completed replication.

Interestingly, all the “early replication” PN3 embryos show peri-NPBs rings of 5MeC, which are fainter or even absent in the “pre-replication” ones (figure 2A). This suggests that the formation of the peri-NPB rings precedes slightly the onset of replication. The 5MeC signal in the paternal pronucleus appears already weaker than in the maternal one prior to replication (figure 2A), while there is little difference for 5hMeC between both pronuclei at this stage (figure 2B). However, 5hMeC patterns change dramatically in the early replication population, with a much stronger signal detected in the paternal pronucleus (figure 2B). 5MeC dynamics are more difficult to assess: the passive demethylation occuring in the maternal pronucleus [11], [12] as well as the increase in pronuclei size make any change difficult to visualize (figure 2A) without proper quantification.

**Figure 3:**
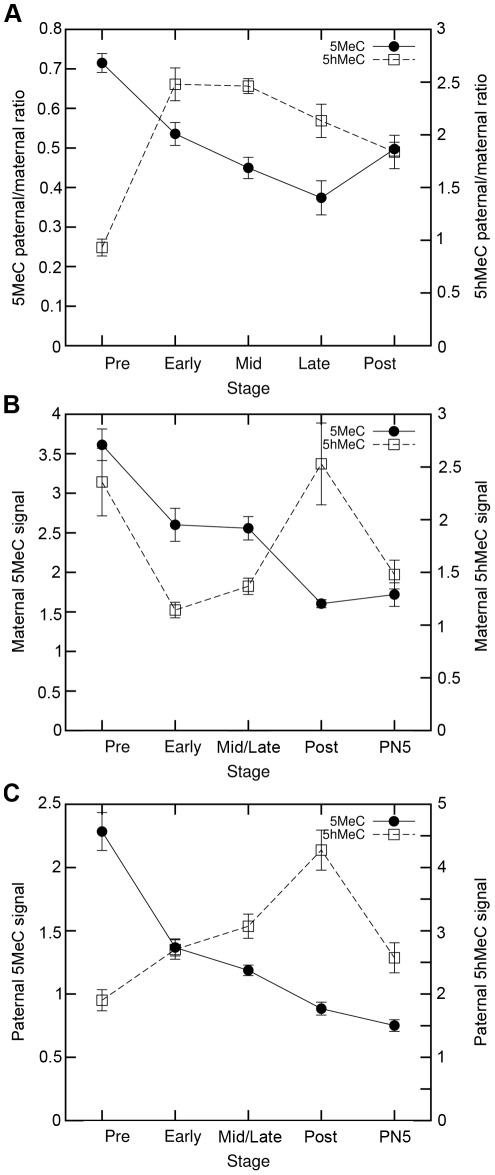
Quantification of 5MeC and 5hMeC over the replication period. Quantification of the global 5MeC and 5hMeC signal was performed and corrected for background and DNA content as described in Material and Methods. A) Comparison of the paternal/maternal ratios obtained for 5MeC and 5hMeC from pre-replication to post-replication. 10 to 27 embryos were quantified for each stage. B) Comparison of 5MeC and 5hMeC dynamics in the maternal pronucleus. Respectively 21 to 46 embryos and 10 to 27 embryos were quantified for each stage. C) Comparison of 5MeC and 5hMeC dynamics in the paternal pronucleus. Respectively 21 to 46 embryos and 10 to 27 embryos were quantified for each stage. Note that different scales were used on all panels for 5MeC and 5hMeC quantifications.

For each embryo, we thus quantified the total DNA methylation content and calculated the paternal/maternal ratio. As can be observed on figure 3A, for 5MeC this ratio decreases the most between the pre-replication and the early replication stage (p-value<10^−4^), with a slighter but constant decrease later on and goes up again after replication (p-value<0.05). The kinetics of the paternal/maternal ratio for 5hMeC is nearly opposite, with a sharp increase between pre-replication and early replication (p-value<10^−11^), no change at mid-replication and a small decrease later on (p-value<0.01 between mid and post-replication).

In order to assess the dynamics of methylation and hydroxymethylation in both pronuclei independently, we used the DNA content as a reference. Since the DNA denaturation necessary for 5MeC and 5hMeC stainings strongly impaired DNA labelling using usual dyes, we used an anti-single stranded DNA antibody. Embryos were again sorted in 4 different replication classes related to the PN stage and hphCG as described above (Table 1). We also analyzed late 1-cell stage embryos at PN5 (28–29 hphCG).

As shown on figure 3B, the normalized DNA methylation content in the maternal pronucleus is divided by around 2 during replication. The most important changes are observed between pre-replication and early replication (p-value<10^−3^) and between mid/late replication and post-replication (p-value<10^−4^). Normalized 5hMeC levels in the maternal pronucleus decrease between pre-replication and early replication (p-value<10^−3^). There is then little change during replication but a sharp and surprising increase after, with a peak post-replication (p-value<10^−3^) followed by an important decrease at the end of the cell cycle (p-value<0.01).

A similar peak is observed in the paternal pronucleus (figure 3C). Indeed normalized 5hMeC levels increase more importantly during replication (from1.90±0.17 prereplication to 2.70±0.15 in early replication; p-value<0.01; and 3.07±0.19 in mid/late replication; p-value<10^−4^), and even more between mid/late replication and postreplication (p-value<10^−3^) before strongly decreasing at the end of the cell cycle (p-value<10^−3^). During the same time, the normalized DNA methylation constantly decreases and is divided by 3, suggesting that passive demethylation cannot be the only mechanism involved in demethylating the paternal DNA, and that active demethylation still takes place during replication.

**Figure 4:**
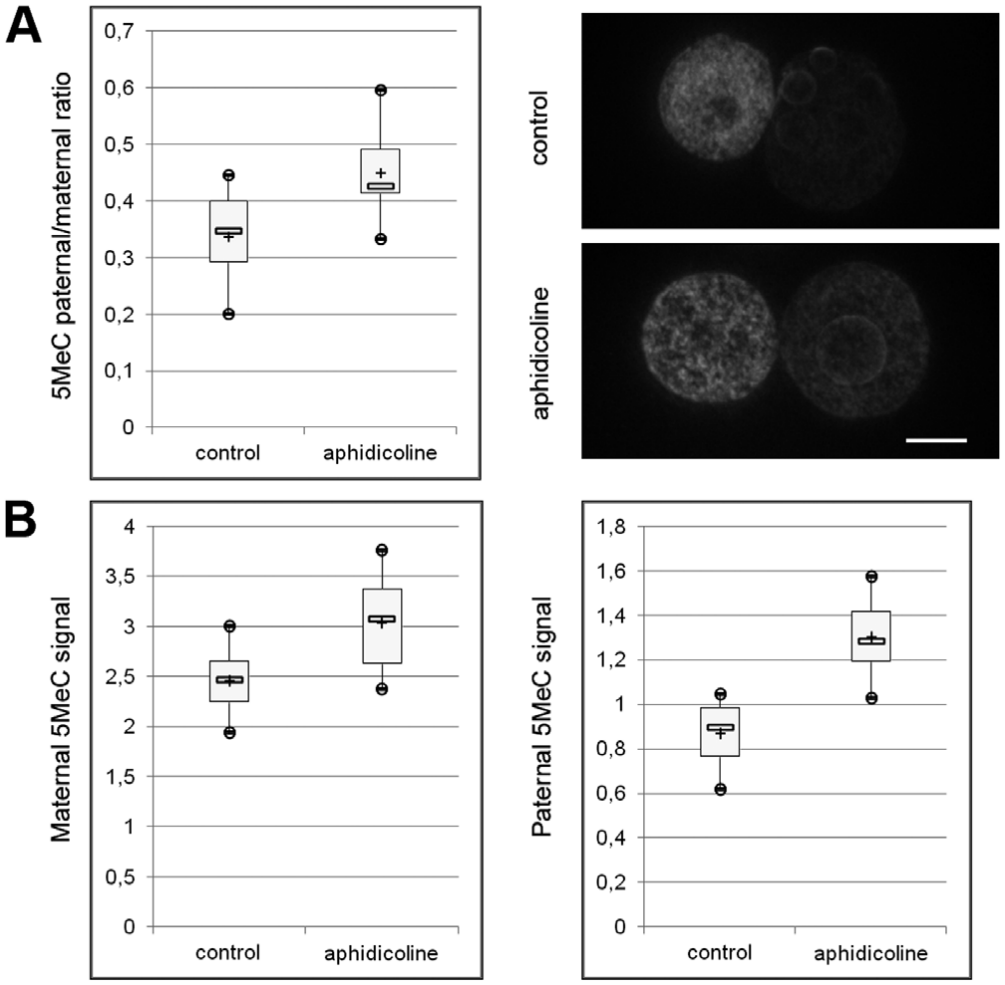
Relationship between DNA demethylation and replication. A) Quantification of the 5MeC paternal/maternal ratio in control (methanol) and aphidicolin treated embryos with representative images (z-projections of 3D-stacks). Images were rotated if necessary to have the maternal PN on the left and the paternal PN on the right. Scale Bar: 10 µm. B) Separate quantification of 5MeC for both pronuclei in control and aphidicolin treated embryos. 4 experiments were performed and a total of 59 (5+17+25+12) control embryos versus 61 (7+18+22+14) aphidicolin treated ones were quantified.

In order to further investigate the relationship between replication and this demethylation, we blocked replication using aphidicolin. As shown on figure 4A, the paternal/maternal ratio at 25 hphCG after aphidicolin treatment is significantly different from that of methanol control embryos (0.45±0.07 vs 0.34±0.08; p-value<10^−3^) but not significantly different from that of prereplication embryos (0.49±0.13; p-value>0.3). We then looked at DNA methylation levels independantly. As shown on figure 4B, an effect of the aphidicolin treatment can be observed in both pronuclei, but is much milder in the maternal pronucleus. It thus appears that in the paternal pronucleus the passive dilution mechanism as well as the active mechanism are blocked following aphidicolin treatment.

## Discussion

In this study, we have analyzed the comparative dynamics of DNA methylation and hydroxymethylation during the first-cell stage in the mouse embryo, both qualitatively, using pericentric heterochromatin and quantitatively, over the replication period.

### 5hMeC as an intermediate of DNA demethylation

Previous studies about the kinetics of hydroxymethylation during the first cell stage have shown that hydroxymethylation remains very low in the maternal pronucleus while it increases in the paternal one, in parallel to demethylation [12], [20]–[23]. While our results regarding the dynamics of 5hMeC in the paternal pronucleus are mostly in agreement with those data, it is not the case in the maternal pronucleus, where we always observe a non negligible signal. Looking at the protocols used, it appears that in several studies [12], [20], [21], [23], the blocking time was increased while the incubation time with the primary antibodies was reduced when compared with standard protocols usually used for 5MeC 3D immunostainings in embryos [10], [34]–[36]. In another study [22], the authors used a protocol similar to ours but with a much lower dilution of the antibody (1∶5000 vs 1∶500) and a signal amplification. In addition, in all of the above mentioned papers, data were obtained with double immunostainings for 5MeC and 5hMeC together.

When we compared these protocols, we found that the modified protocols affect the results: 5hMeC signal is of lower quality and thus appears more prominent in the paternal pronucleus (Figure S1). We observed that the signal ratio between the maternal and paternal pronuclei was modified when the antibody concentration went too low (1∶2000 dilution; data not shown), because some of the signal was lost especially in the maternal pronucleus. It is therefore not surprising that, with a higher antibody dilution (1∶5000) Ruzov and collaborators only observed paternal pronucleus staining: even if they used an amplification system, it cannot restore lost signal [22]. On the other hand, using lower antibody dilutions (1∶100; [21]) might saturate the signal in the paternal pronucleus and alter the paternal/maternal ratio. To minimize these problems, we chose an intermediate dilution (1∶500; as in [12]) that provided reproducible results in terms of signal distribution and intensity. Finally, the use of double immunostainings also increased the difference in intensity between both pronuclei, in comparison to single immunostaining (Figure S2). We think this might be due to antibody competition, caused by steric hindrance when both marks are found in the vicinity of each other. It therefore appears that 5hMeC staining is very sensitive to the protocol used and this could explain the differences between our study and previous ones.

Following its rediscovery, 5hMeC has rapidly been proposed as an intermediate in the active demethylation process [18]. It also fits with the patterns of 5hMeC observed in previous studies [12], [20]–[23], [30]. Indeed, in the paternal pronucleus, all studies, including ours, show an increase of 5hMeC concomitant with the decrease of 5MeC, which is in agreement with the hypothesis that 5MeC is converted to 5hMeC. In the maternal pronucleus, it has been suggested that maternal demethylation happens by dilution, i-e through a passive replication-dependent mechanism. This hypothesis is supported by data from Rougier [11] and Inoue [12] showing the progressive disappearance of 5MeC from metaphase chromosomes during the first embryonic division, as well as by our own data showing that the 5MeC/DNA ratio is divided by 2 during replication in the maternal pronucleus. The absence of 5hMeC in the maternal pronucleus would be in agreement with this hypothesis.

Some data however do not fit in this perfect picture. First, our results do not only show the presence of 5hMeC in the maternal pronucleus all along the cell cycle, but also its strong increase during the replication phase. Second, a closer look at 5MeC and 5hMeC kinetics in the paternal pronucleus shows that the increase in 5hMeC observed between late and post-replication is much more important than we would expect from the 5MeC decrease at the same time. Third, at the end of the cell cycle, we observe an important enrichment of 5hMeC on the major satellites in the paternal pronucleus, unrelated to any decrease of the 5MeC signal. Finally, we show that at least part of the active demethylation in the paternal pronucleus is dependent on replication, while Wossidlo and collaborators have shown that 5hMeC does not depend on replication [21].

Several non exclusive hypotheses could explain these results. First, 5hMeC could participate in passive demethylation too. This has already been proposed by Tahiliani and collaborators [18], based on the fact that Dnmt1 recognizes poorly 5hMeC [37]. However, while this would explain in part the 5hMeC increase in the maternal pronucleus it does not explain the other observations.

Second, the demethylation process could be more complex than it appears at first glance, with the coexistence of passive and active demethylation as well as of methylation in both pronuclei. This would require the presence of an active Dnmt. Several studies have shown that the maintenance Dnmt1 [38]–[40] and the *de novo* Dnmt3A [41], are expressed in both pronuclei of mouse zygotes. Some methylation could therefore take place in the maternal pronucleus as well as in the paternal pronucleus. This hypothesis is supported by the reinforcement of 5MeC, as well as of 5hMeC, that we observe on peri-NPB sequences in the paternal pronucleus between PN4 and PN5. Moreover, methylation of the paternal genome has already been shown in 1-cell embryos of rabbit [42] and bovine [35] using 5-Azacytidine, a DNA methylation inhibitor. Preliminary experiments we performed in the mouse embryos suggest that methylation at 29 hphCG, but not at 25 hphCG, is indeed reduced in both pronuclei after treatment with 5-Azacytidine (data not shown), but this would need further examination.

### Other functions for 5hMeC?

Results from several studies [12], [20], [22] have shown a persistence of 5hMeC during preimplantation development, while other studies have emphasized the relatively strong presence of 5hMeC in pluripotent cells [22], [31]. Inoue and collaborators have shown that 5hMeC but also its derivates (5-formylcytosine and 5-carboxylcytosine) are lost by dilution through the cell cycles [12], [30]. This 5hMeC persistence suggests that it plays a role on its own.

In agreement with this hypothesis, we show that the patterns of 5MeC and 5hMeC on the pericentric heterochromatin are not exactly complementary, unlike what had been suggested by other authors [20], [21]. 5hMeC appears enriched only on a fraction of the pericentric heterochromatin in the maternal pronucleus, which probably represents a subset of the chromosomes (in mouse all chromosomes, except for the Y, carry pericentric heterochromatin). This in accordance with images from Inoue and collaborators [12], on which only 6 out of 20 maternally inherited chromosomes show a strong 5hMeC staining. Interestingly, while 5MeC does not show any enrichment on the maternal heterochromatin during most of the cell cycle, a partial enrichment is detected on those sequences at the end of the cell cycle (PN5 and beyond), probably also on a subset of chromosomes. This had already been noted on metaphase chromosomes by Rougier and collaborators [11], and is also visible on images from Inoue [12], where a colocalization of 5MeC and 5hMeC can be observed on some chromosomes. It would be interesting to determine which chromosomes show enrichment for either one or both marks on its pericentric heterochromatin.

Coexistence of both marks is also observed at the end of the cell cycle in the paternal pronucleus, not only on pericentric heterochromatin but also on other sequences localizing around the NPBs. Those could correspond to other repetitive sequences such as LINEs, in agreement with results from Iqbal [20], or the ribosomal DNA [43]. Moreover, as this coexistence of 5MeC and 5hMeC can also be observed in ES cells [31], [44] but not in somatic adult cells where 5hMeC is mostly absent from repeat sequences [45], we can hypothesize that it may represent a specific epigenetic state of embryos or embryonic cells.

Finally, the dynamics of 5hMeC lead to a peak of this mark in both pronuclei after the completion of replication. Since 5hMeC has been related to the regulation of gene expression in ES cells [44], [46], [47], it is possible that 5hMeC plays a role in the minor activation of the embryonic genome, which occurs simultaneously with this peak [32], [48]. Further studies are necessary to assess a possible role of 5hMeC in this process, as well as in the major activation which occurs at the 2-cell stage in mouse.

In conclusion, the conversion of 5MeC to 5hMeC could indeed be an intermediate step in demethylation during early development, but increasing evidences indicate that it could be as well a conversion from a repressive epigenetic mark to another, more permissive for transcription.

## Materials and Methods

### Ethics Statement

Animal care and handling were carried out according to European regulations on animal welfare. NB and JS both have the authorization to work with laboratory animals from the departmental veterinary regulatory services (N° 78-95 and N° 78-137, respectively). This work has been approved by the local ethics committee (agreement 11/048 from the Comethea Jouy-en-Josas/AgroParisTech).

### Embryo collection and culture

All products are from Sigma-Aldrich, France unless otherwise stated.

Embryos were produced by natural fertilization of C57/CBA F1 mice. Superovulation was induced by injection of pregnant mare serum gonadotropin (PMSG, Intervert, 5 UI) followed by injection of human chorionic gonadotropin (hCG, Intervert, 5 UI) 48 hours later. Female mice were then mated with C57/CBA F1 males. Fertilization occurred at about 12 hours after hCG injection which was used as reference point for embryonic development (hours post-hCG i.e., hphCG). Fertilized eggs were collected at 18–19 hphCG from the ampulla in M2 medium after a brief treatment with 1 mg/ml of hyaluronidase in phosphate buffered saline (PBS, pH7.5, AMRESCO, Solon, OH) to separate them from the surrounding follicular cells.

After collection, embryos were transferred to M16 medium and kept in an incubator at 37°C under 5% CO_2_. Depending on the experiment set, various compounds could be added to the M16 medium.

All experimental sets contained embryos from six to ten different mice. All experiments were repeated at least twice.

### Assessment of replication

To avoid cross-reactivity with anti 5hMeC or 5MeC antibodies, labeling of replication was performed either by BrdU incorporation or by DIG-dUTP micro-injection.

In the first case, embryos were incubated in the presence of BrdU (100 µM in M16 medium) for 30 minutes in an incubator at 37°C under 5% CO_2_ and subsequently fixed with 4% paraformaldehyde (PFA, EMS, Hatfield, PA) in PBS overnight at 4°C. These embryos incubated with BrdU were later used for anti-5hMeC and anti-BrdU double immunostainings. In the second case, DIG-dUTP (40 µM in Pipes/KCl, Roche, Switzerland) was injected in embryos using a Nikon inverted microscope with Narishige micromanipulators and an Eppendorf microinjector. Injections were performed between 22 and 25 hphCG. After injection embryos were placed in M16 in the incubator at 37°C under 5% CO_2_ for 30 minutes and subsequently fixed with 4% paraformaldehyde (PFA) in PBS overnight at 4°C.

Embryos micro-injected with DIG-dUTP were later used for anti-5MeC and anti-DIG double immunostainings.

### Aphidicolin experiments and controls

Embryos were collected at 18 hphCG. As a first control, some embryos were incubated in the presence of BrdU for 30 minutes at 19 hphCG and fixed in PFA 4% overnight at 4°C to check that replication had not started. Immunostaining was therefore performed on those control embryos with an anti-BrdU antibody. The rest of the embryos were incubated in M16 supplemented with either Aphidicolin (10 µg/ml, 1/400 from a 4 mg/ml stock solution diluted in methanol) or methanol (1/400 v/v) as a second control, starting from 19 hphCG. The medium was changed every 2 hours to avoid temperature-linked degradation of the drug. At 23 hphCG, a group of embryos from both batches were incubated with BrdU, fixed in PFA 4% overnight at 4°C and later stained with an anti-BrdU antibody to verify the absence of replication in the aphidicolin treated ones. The remainder of the embryos (aphidicolin treated and methanol controls) were fixed at 25 hphCG, in PFA 4% overnight at 4°C and processed for 5MeC immunostaining.

### Immunofluorescence and Mounting

Embryos were fixed with 4% PFA in PBS overnight at 4°C and permeabilized with 0.5% Triton X-100 (30 min, Room Temperature (RT)) after several washes with 0.05% Tween-20 in PBS. They were blocked with 2% bovine serum albumin (BSA) in PBS for 1 h. Embryos were then incubated in 2N HCl solution at 37°C for 1 hour. Incubation with various combinations of the following primary antibodies, was performed overnight at 4°C: anti-5-methylcytosine (5MeC, mouse monoclonal Eurogentec, BI-MECY1000), anti-5-hydroxymethylcytosine (5hMeC, rabbit polyclonal Active Motif 39769), anti-BrdU (mouse monoclonal Becton Dickinson 347580) and anti-digoxigenin (DIG, sheep polyclonal, Roche 11333089001) diluted in 2% BSA-PBS at 1∶500, 1∶500, 1∶100 and 1∶200, respectively.

When no DNA immunostaining was performed, after two washes with 0.05% Tween-20 in PBS (15 min each, RT), embryos were incubated with the secondary antibodies, coupled with Fluorescein (FITC), Rhodamine (TRITC) or Cyanine 5 (Cy5) (Jackson Immunoresearch, West Grove, PA) and diluted in 2% BSA-PBS at 1∶200, during 1 hr (RT). They were extensively rinsed again to remove excess of antibodies and briefly postfixed (2% PFA-PBS, 20 min, RT).

When DNA immunostaining was performed, after two washes with 0.05% Tween-20 in PBS (15 min each, RT), embryos were postfixed (2% PFA-PBS, 20 min, RT) and subsequently incubated in 4% non fat milk-0.1% Tween-PBS for 3 hours. Incubation with the primary anti-DNA single stranded specific antibody (ssDNA, mouse IgM monoclonal, Millipore clone F7-26) diluted in 4% non fat milk-0.1% Tween-PBS at 1∶10 was performed for 36 hours at 4°C. After two washes with 0.05% Tween-20 in PBS (15 min each, RT), embryos were incubated with the secondary antibodies, coupled with FITC, TRITC or Cy5 (Jackson Immunoresearch, West Grove, PA) and diluted in 2% BSA-PBS at 1∶100, during 1 hr (RT). They were extensively rinsed again to remove excess of antibodies and briefly postfixed (2% PFA-PBS, 20 min, RT).

The embryos were finally deposited on slides and mounted under a coverslip with citifluor (Citifluor Products, Canterbury, UK).

Examples of co-immunostainings obtained with the ssDNA and the 5hMeC antibodies are shown on Figure S3.

### Protocol controls

To ensure that the signals detected for 5hMeC and 5MeC using our protocol were specific, antibodies against anti-5-hydroxymethylcytosine (5hMeC, rabbit polyclonal Active Motif 39769) or anti-5-methylcytosine (5MeC, mouse monoclonal Eurogentec, BI-MECY1000) were preincubated with either 1 µM 2′-deoxy-5′-methylcytidine-5′-triphosphate (dm^5^CTP, Fermentas) or 1 µM 2′-deoxy-5′-hydroxymethylcytidine-5′-triphosphate (Hydroxymethyl-dCTP, BIOLINE). Immunostaining experiments were then performed as described above using the preincubated anti-5-hydroxymethylcytosine or anti-5-methylcytosine antibodies. Results are presented in Figure S4.

### 3D ImmunoFISH

Embryos were fixed with 2% PFA in PBS 20 minutes at room temperature (RT) and permeabilized with 0.5% Triton X-100 (30 min, RT) after several washes with 0.05% Tween-20 in PBS. They were blocked with 2% bovine serum albumin (BSA) in PBS for 1 h. Embryos were then incubated in 4N HCl solution at RT for 20 minutes. Incubation with primary antibody either anti-5-methylcytosine (5MeC, mouse monoclonal Eurogentec, BI-MECY1000), or anti-5-hydroxymethylcytosine (5hMeC, rabbit polyclonal Active Motif 39769) diluted in 2% BSA-PBS at 1∶500 was performed overnight at 4°C. After two washes with 0.05% Tween-20 in PBS (15 min each, RT), embryos were incubated with the secondary antibodies, coupled with FITC (Jackson Immunoresearch, West Grove, PA) and diluted in 2% BSA-PBS at 1∶200, during 1 hr (RT). They were extensively rinsed again to remove excess of antibodies and briefly postfixed (2% PFA-PBS, 20 min, RT). The remainder of the zona pellucida was removed using acidic tyrode (40 seconds, RT) and embryos were mounted on Superfrost slides after a brief rinse in PBS, and postfixed again in PFA 4% 30 minutes at RT. A new permeabilization was performed (Triton X100 0.5%, 30 minutes, RT), followed by a brief wash in 2× SSC pH6.3 and a RNAse A treatment (200 µg/ml in 2× SSC pH6.3; 30 minutes, 37°C). Slides were then equilibrated in hybridization buffer (50% formamide, SCC 2×, Denhardt 1×, 40 mM NaH2PO4, 10% dextran sulfate) for 1 h at RT. Both probes (diluted in hybridization buffer) and slides were separately denatured by heating 10 minutes at 85°C, then the major satellites probes were applied on the embryos for incubation at 37°C overnight. After two 5 minutes washes in 2× SSC pH6.3 at 42°C, a last postfixation was performed (PFA 2%, 15 minutes, RT) and the slides were mounted with citifluor (Citifluor Products, Canterbury, UK).

Major satellites probes were prepared by PCR on genomic mouse DNA using the 2 following primers 5′-CATATTCCAGGTCCTTCAGTGTGC-3′ and 5′-CACTTTAGGACGTGAAATATGGCG-3′ and Cy5-labeling by random priming (Invitogen Kit, Ref 18095-011).

### Fluorescence Microscopy and Image Analysis

For immunostainings experiments, FITC, TRITC and Cy5 signals were recorded with a Zeiss ApoTome structured illumination system using an oil-immersion objective (Plan Apochromatic 63×, n.a.1.4) and 470 nm, 530 nm or 625 nm LEDs. The distance between two consecutive optical sections was 0.27 µm.

For ImmunoFISH experiments, FITC and Cy5 signals were recorded with a Zeiss LSM 510 confocal laser scanning microscope equipped with an oil-immersion objective (Plan Apochromatic 63×, n.a.1.4) and lasers at 488- and 633-nm wavelengths. Entire embryos were scanned with 0.37 µm distance between light optical sections.

Quantitative analysis was performed using the ImageJ software. Projection summing intensities of all selected slices was performed independently on both pronuclei. A region of interest was then drawn around each pronucleus and the total fluorescence intensity was measured for 5MeC or 5hMeC and ssDNA stainings. Background correction was applied for all signals. ssDNA staining was used for normalization of paternal/maternal ratios and to obtain an estimation of 5MeC or 5hMeC kinetics in both pronuclei independently.

Graphs were obtained with the Gnuplot software and a specific macro, developed by Claude Monteil (INP/ENSAT Toulouse; http://www.inp-toulouse.fr/fr/espace-tice/excel-interactif.html#affichage) was used to generate boxplots with the Excel software (Microsoft). Figures were built-up using Adobe Photoshop CS4 software.

### Statistical Analysis

The staining intensity ratios at the different stages were compared with a Mann–Whitney–Wilcoxon test at level 5% using the R statistical software package [49].

## Supporting Information

Figure S1 **Comparison of two immunostaining protocols used to detect 5hMeC.** Images were acquired as 3D stacks and single representative sections (A/B) as well as z-stack projections (A′/B′) are shown. A and A′) Images obtained using the protocol from Iqbal *et al.*, 2011 [20] (n = 13). B and B′) Images obtained using the procedure commonly used for 5MeC immunostaining (n = 25). Scale Bar: 10 µm.
(TIF)

Figure S2 **Effect of double 5MeC/5hMeC immunostaining.** Z-stack projections of embryos stained either with an anti-5hMeC antibody alone (single immunostaining; n = 21) or with both an anti-5hMeC and an anti-5MeC antibody (double immunostaining; n = 22). PB: Polar Body; Scale Bar: 10 µm. Maternal PN (m) and paternal PN (p) clearly do not show the same type of staining in both cases as underlined by the quantification of the paternal/maternal ratio for 5hMeC.
(TIF)

Figure S3 **5hMeC and DNA stainings.** Representative z-section images of maternal PN (left) and paternal PN (right) in 1-cell embryos at the PN3 and PN5 stages with double immunostainings for single-stranded DNA (DNA panel, red on the merge panel) and 5hMeC (5hMeC panel, green on the merge panel). Scale Bar: 5 µm.
(TIF)

Figure S4 **Specificity of 5hMeC staining.** Z-stack projections of embryos stained with either an anti-5MeC (A,B) or an anti-5hMeC antibody (C,D) that were preincubated with methyl-dCTP (A,C) or with hydroxymethyl-dCTP (B,D), before the immunostaining procedure. Around 10 embryos were analyzed per group. Scale Bar: 10 µm.
(TIF)
