# Supplementary figures and images for: 5-Methylcytosine and 5-Hydroxymethylcytosine Spatiotemporal Profiles in the Mouse Zygote
Source: PLoS One. 2012 May 31;7(5):e38156. doi: 10.1371/journal.pone.0038156 (PMC3364968; doi:10.1371/journal.pone.0038156)

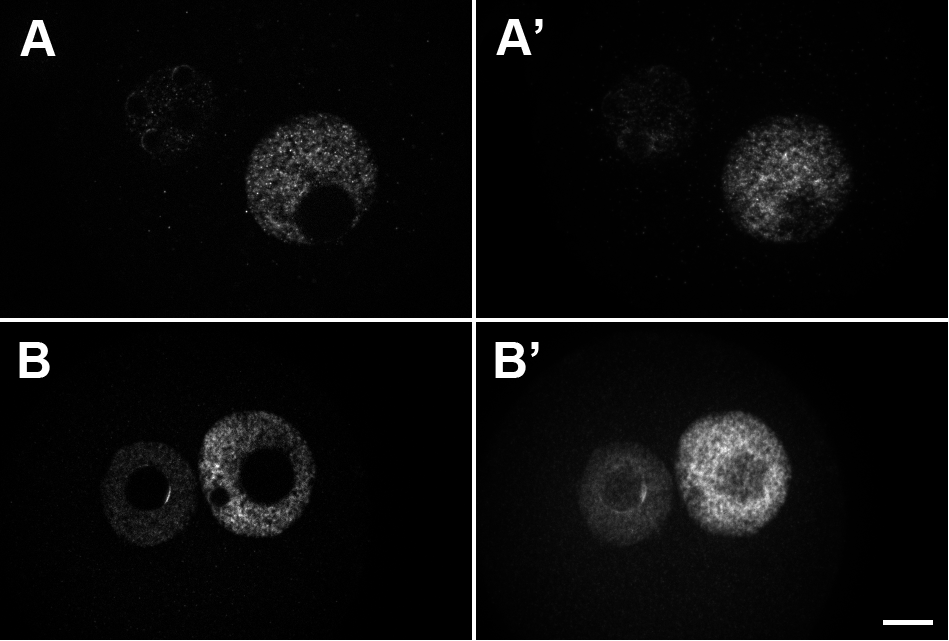

Supplement: Figure S1 — Comparison of two immunostaining protocols used to detect 5hMeC. Images were acquired as 3D stacks and single representative sections (A/B) as well as z-stack projections (A′/B′) are shown. A and A′) Images obtained using the protocol from Iqbal et al., 2011 [20] (n = 13). B and B′) Images obtained using the procedure commonly used for 5MeC immunostaining (n = 25). Scale Bar: 10 µm. (TIF) [file pone.0038156.s001.tif]

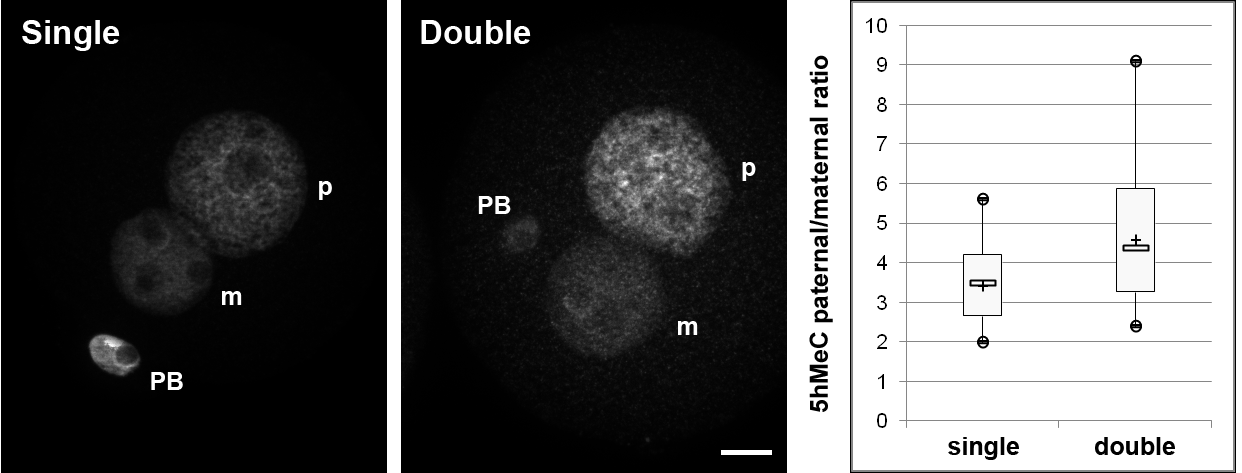

Supplement: Figure S2 — Effect of double 5MeC/5hMeC immunostaining. Z-stack projections of embryos stained either with an anti-5hMeC antibody alone (single immunostaining; n = 21) or with both an anti-5hMeC and an anti-5MeC antibody (double immunostaining; n = 22). PB: Polar Body; Scale Bar: 10 µm. Maternal PN (m) and paternal PN (p) clearly do not show the same type of staining in both cases as underlined by the quantification of the paternal/maternal ratio for 5hMeC. (TIF) [file pone.0038156.s002.tif]

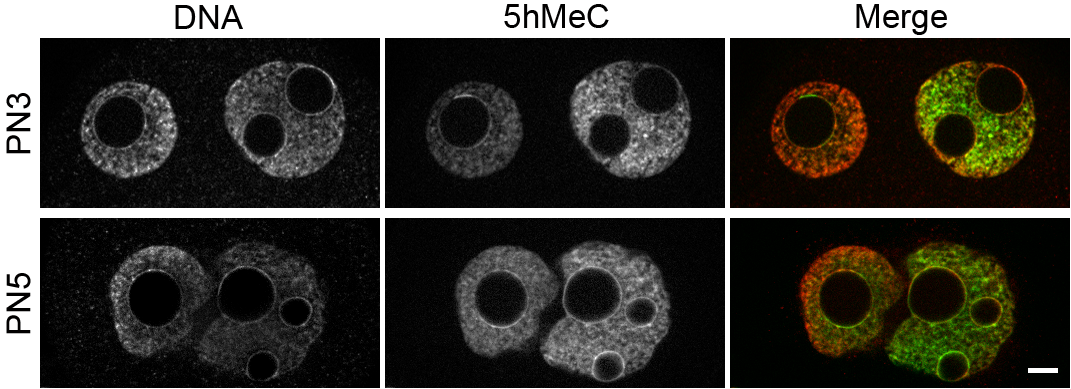

Supplement: Figure S3 — 5hMeC and DNA stainings. Representative z-section images of maternal PN (left) and paternal PN (right) in 1-cell embryos at the PN3 and PN5 stages with double immunostainings for single-stranded DNA (DNA panel, red on the merge panel) and 5hMeC (5hMeC panel, green on the merge panel). Scale Bar: 5 µm. (TIF) [file pone.0038156.s003.tif]

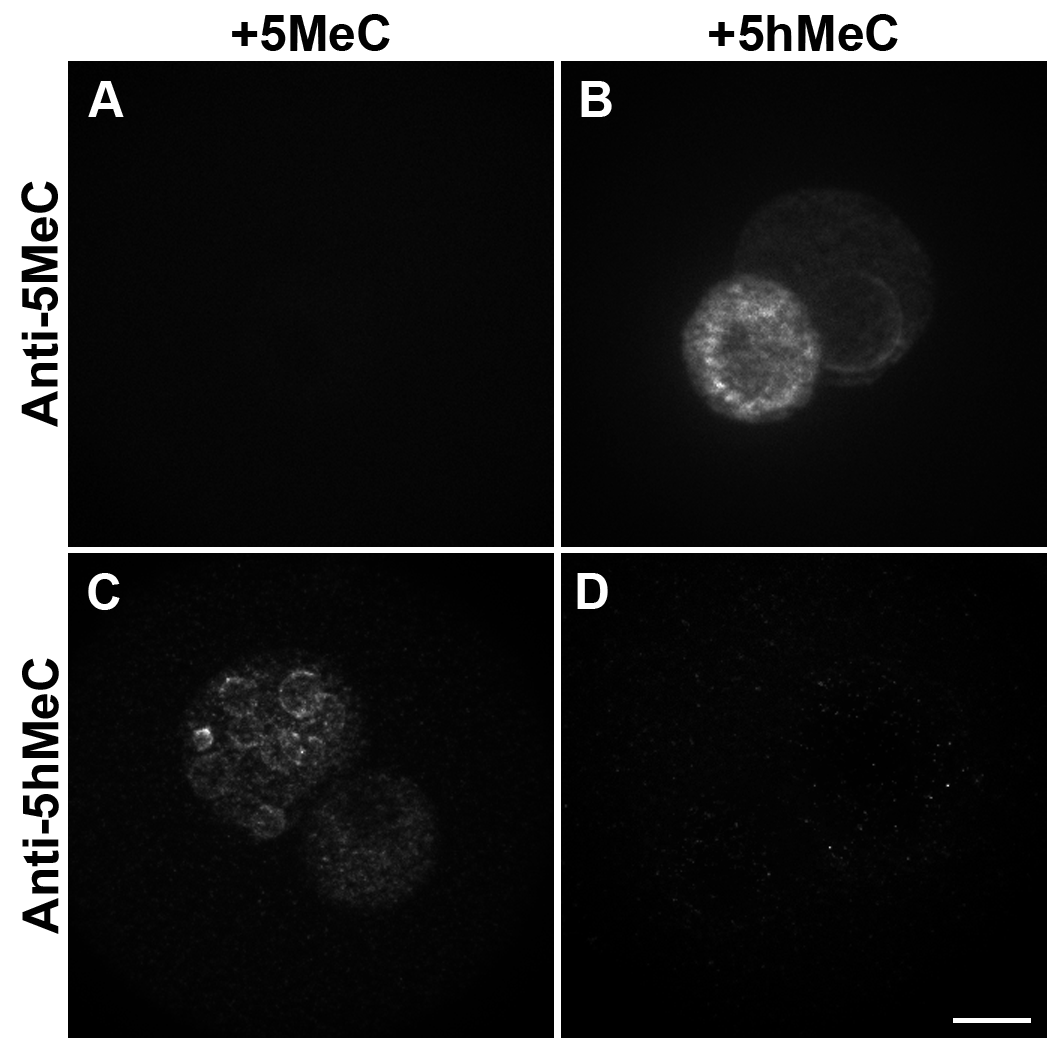

Supplement: Figure S4 — Specificity of 5hMeC staining. Z-stack projections of embryos stained with either an anti-5MeC (A,B) or an anti-5hMeC antibody (C,D) that were preincubated with methyl-dCTP (A,C) or with hydroxymethyl-dCTP (B,D), before the immunostaining procedure. Around 10 embryos were analyzed per group. Scale Bar: 10 µm. (TIF) [file pone.0038156.s004.tif]
